# Supplementary material for: Sustainable palm oil certification inadvertently affects production efficiency in Malaysia
Source: Commun Earth Environ. 2025 Mar 12;6(1):200. doi: 10.1038/s43247-025-02150-2 (PMC11903298; doi:10.1038/s43247-025-02150-2)
Supplement: Supplementary file 2 — Supplementary Material [file 43247_2025_2150_MOESM2_ESM.pdf]

Supplementary Table 1: Pearson Correlations

Sample 1	Mean	S.D.	Max.	Min.	1	2	3	4	5	6	7	8	9	10	11	12	13
1. Oil Palm Plantation Efficiency	0.753	0.188	1.000	0.185													
2. Vegetation Health	0.699	0.057	0.896	0.361	0.506***												
3. Groundwater Availability	0.986	0.004	0.994	0.962	0.263***	0.454***											
4. Soil Visibility	-0.749	0.043	-0.499	-0.899	-0.512***	-0.946***	-0.476***										
5. Palm Oil Price	3420.571	970.539	5045.500	1940	0.094*	0.004	0.078*	-0.003									
6. Year 2017	0.143	0.350	1.000	0.000	-0.095*	-0.186***	-0.330***	0.222***	-0.425***								
7. Year 2018	0.143	0.350	1.000	0.000	0.012	0.230***	0.209***	-0.245***	-0.623***	-0.167***							
8. Year 2019	0.143	0.350	1.000	0.000	-0.138***	-0.083*	-0.086*	0.090*	-0.173***	-0.167***	-0.167***						
9. Year 2020	0.143	0.350	1.000	0.000	0.048	-0.155***	-0.101**	0.135***	0.145***	-0.167***	-0.167***	-0.167***					
10. Year 2021	0.143	0.350	1.000	0.000	0.046	0.083*	0.039	-0.055	0.684***	-0.167***	-0.167***	-0.167***	-0.167***				
11. Year 2022	0.143	0.350	1.000	0.000	-0.148***	0.046	0.137***	-0.065	0.223***	-0.167***	-0.167***	-0.167***	-0.167***	-0.167***			
12. Year 2023	0.143	0.350	1.000	0.000	0.275***	0.064	0.132**	-0.083*	0.170***	-0.167***	-0.167***	-0.167***	-0.167***	-0.167***	-0.167***		
13. Self-Produced	0.221	0.415	1.000	0.000	0.170***	0.178***	0.066	-0.172***	0.000	0.000	0.000	0.000	0.000	0.000	0.000	0.000	
14. Outsourced	0.305	0.461	1.000	0.000	-0.059	-0.071	0.038	0.091*	0.000	0.000	0.000	0.000	0.000	0.000	0.000	0.000	-0.629***

N = 665; ∗ $p < 0.05$; ∗∗ $p < 0.01$; ∗∗∗ $p < 0.001$ (two-tailed)

Sample 2	Mean	S.D.	Max.	Min.	1	2	3	4	5	6	7	8	9	10	11	12	13
1. Oil Palm Plantation Efficiency	0.736	0.206	0.999	0.004													
2. Vegetation Health	0.680	0.053	0.765	0.306	0.569***												
3. Groundwater Availability	0.986	0.004	0.993	0.957	0.149**	0.222***											
4. Soil Visibility	-0.737	0.039	-0.485	-0.794	-0.560***	-0.970***	-0.254***										
5. Palm Oil Price	3420.571	971.255	5045.500	1940.000	0.022	0.136**	-0.186**	-0.125*									
6. Year 2018	0.143	0.350	1.000	0.000	-0.123*	-0.048	0.134**	0.038	-0.623***								
7. Year 2017	0.143	0.350	1.000	0.000	0.205***	-0.166**	0.025	0.188**	-0.425***	-0.167**							
8. Year 2019	0.143	0.350	1.000	0.000	-0.230***	-0.072	0.067	0.060	-0.173**	-0.167**	-0.167**						
9. Year 2020	0.143	0.350	1.000	0.000	-0.022	-0.072	-0.341***	0.079	0.145**	-0.167**	-0.167**	-0.167**					
10. Year 2021	0.143	0.350	1.000	0.000	-0.044	-0.030	-0.177**	0.059	0.684***	-0.167**	-0.167**	-0.167**	-0.167**				
11. Year 2022	0.143	0.350	1.000	0.000	-0.116*	0.188**	0.169**	-0.207***	0.223***	-0.167**	-0.167**	-0.167**	-0.167**	-0.167**			
12. Year 2023	0.143	0.350	1.000	0.000	0.331***	0.200***	0.123*	-0.217***	0.170**	-0.167**	-0.167**	-0.167**	-0.167**	-0.167**	-0.167**		
13. Self-Produced	0.104	0.306	1.000	0.000	0.062	0.060	0.081	-0.056	0.000	0.000	0.000	0.000	0.000	0.000	0.000	0.000	
14. Outsourced	0.229	0.421	1.000	0.000	0.098	0.148**	0.050	-0.158**	0.000	0.000	0.000	0.000	0.000	0.000	0.000	0.000	-0.186**

N = 336; ∗ $p < 0.05$; ∗∗ $p < 0.01$; ∗∗∗ $p < 0.001$ (two-tailed)
